# The role of community factors in predicting depressive symptoms among Chinese workforce: a longitudinal study in rural and urban settings

**DOI:** 10.1186/s12889-022-13647-2

**Published:** 2022-07-27

**Authors:** Wanlian Li, Guanghan Gao, Fei Sun, Lin Jiang

**Affiliations:** 1 Emergency Management Teaching and Research Department, Guangdong Institute of Public Administration, No. 3 Jianshe Ave., Yuexiu District, Guangzhou, Guangdong China; 2 Zhou Enlai School of Government, Nankai University, No. 38 Tongyan Road, Jinnan District, Tianjin, China; 3 School of Social Work, Michigan State University, 150 Baker Hall, 655 Auditorium Rd., East Lansing, MI USA; 4 School of Social Work, University of Texas Rio Grande Valley, 1201 W. University Drive, TX Edinburg, USA

**Keywords:** Disparities, Workforce, Depressive symptoms, Community networks

## Abstract

**Background:**

The dual urban–rural division system in China has led to distinguishes in economic development, medical services, and education as well as in mental health disparities. This study examined whether community factors (community cohesion, supportive network size, foreseeable community threat, and medical insurance coverage) predict the depressive symptoms of Chinese workers and how community factors may work differently in rural and urban settings.

**Methods:**

This secondary data analysis was conducted using data from the 2014 and 2016 China Labor-force Dynamics Survey (CLDS). The sample of this study includes 9,140 workers (6,157 rural labors and 2,983 urban labors) who took part in both the 2014 and 2016 CLDS. This study discusses the relation between community factors and depressive symptoms of Chinese workers by correlation analysis and regression analysis. All analyses were conducted using SPSS 24.0.

**Results:**

The results indicate that rural workers have higher levels of depressive symptoms than urban workers. Medical benefits coverage predicts depressive symptoms of rural workforces (B = -0.343, 95%CI = -0.695 ~ 0.009, *p* < . 10), and community supportive network size predicts depressive symptoms of urban workforces (B = -.539, 95%CI = -0.842 ~ 0.236, *p* < . 01).

**Conclusions:**

Policymakers may address depressive symptoms of rural labor through improved coverage of medical benefits. In urban areas, efforts can be made to strengthen community supportive network for the urban labor force.

## Background

The prevalence of depressive symptoms has increased over the past decades globally [1]. One study using the China Family Panel Studies (CFPS), which covers 40,000 respondents from 25 Chinese provinces, found that rate of depressive symptoms reached 37.86% [1]. Depression poses serious social challenges given its close relation with disability, disease burden, physical wellbeing, human capital accumulation, medical cost, and economic loss [1–3]. Additionally, the regional disparity in depressive symptoms in China is noteworthy: in rural areas, the prevalence of depressive symptoms is 41.21%, much higher than the rate in urban areas 31.49% [1]. Furthermore, urban residents have 3.1% lower likelihood of having depressive symptoms than rural residents [1]. Therefore, it is necessary to examine the disparities of depressive symptoms between the rural and urban contexts and related factors.

### Theoretical framework

The field theory [4] provides a framework to examine factors that influence an individual in social situations [5]. It poses those human behaviors result from the interaction between the individual and the total environment (field). The interaction between individuals and the surrounding environment influences mental health in two ways: (1) reconstructing personal living space and (2) forming community-related factors that affect an individual’s mental health [6]. Community factors can influence individual mental health through structural factors (e.g., community resources) and relational factors (e.g., community relationships). Rural and urban communities have shown differences in structural and relational dimensions [6]. Thus, both community factors and urban-rural differences in community factors are examined in this study.

### Individual factors for depressive symptoms

Existing literature has highlighted an array of demographic and socioeconomic factors on depressive symptoms. Lower-income, lower education, and unemployment have been associated with depressive symptoms [7–9], while higher depressive symptoms tend to be more common among women and young people [10–12]. The influence of education on depressive symptoms is mixed, as literature indicated that both educated and relatively less education people exhibit depressive symptoms [8]. Growing research is interested in uncovering the environmental influence on workers’ depressive symptoms.

### Community factors for depressive symptoms

#### Community cohesion

Current findings related to community cohesion and depressive symptoms are mixed and limited. Previous research has indicated that older Chinese population with high community cohesion reported fewer depressive symptoms [13, 14]. However, Yamaguchi, Inoue, Shinozaki, Saito, Takagi, Kondo, & Kondo [15] pointed out that community cohesion was not associated with depressive symptoms. Similarly, Haseda et al. [16] also found that the influence of community cohesion on depressive symptoms was not significant.

#### Supportive network size

Previous research has demonstrated that participants with higher social support, especially support from friends and neighbors [17] reported fewer depressive symptoms [8, 18]. Analyzed data from a national survey in Australia, Werner-Seidler et al. [19] found that people who interacted with friends less than once a month were 2.19 times more likely to have depressive symptoms, and those without family support had a 3.47 times greater chance of having depressive symptoms.

#### Foreseeable community threat

People perception of the community shapes their understanding of community risks [20]. More than 50% of men think their community is at risk, and less than half of women share similar perceptions [21]. Arthur et al. [22] found that people with higher perceived community insecurity are more prone to an unhealthy disease. However, Moore et al. [23] found that when a person feels safer in the community, the less their depressive symptoms. Similarly, Flórez et al. [24] indicated that the more a person perceives security in their neighborhood, the less their psychological distress.

#### Medical insurance coverage

Medical insurance coverage is considered a community factor because it is essentially a product of the inequalities in medical benefits coverage between rural and urban China [25–27]. Previous studies pointed out the positive effect of medical insurance coverage on health outcomes, which included alleviating depressive symptoms [28]. Also, Chiu & Yang [29] demonstrated a significant relationship between the level of medical insurance and psychological conditions. Furthermore, Tang et al. [30] indicated that urban older adults in China with higher medical insurance coverage reported significantly fewer depressive symptoms than rural older adults.

Existing research has alluded to the significant influence of community factors on people's depressive symptoms. However, empirical research related to the influence of community factors on depressive symptoms among Chinese workers is rare. The first research question of this study is to identify the impact of different community factors on depressive symptoms among these Chinese labor force.

### Urban–rural differences in community factors

German sociologist Tonnies [31] mentions the difference between community and society in his book "Community and Society". In his opinion, the traditional countryside is the typical representative of the community, and the commercialized city is the representative of society. According to Tonnies, rural “communities” and urban “societies” have completely different organizational foundations and characters. In urban "society", people's relationships are based on individual independence and personal reason and contracts and laws. In rural "communities", people are based on a shared history, traditions, beliefs, customs, and trust, forming an interpersonal relationship with intimacy, mutual trust, and watchfulness. The community in this article refers to various social associations and social groups composed of people living in a specific place and a regional living community consisting of multiple social activities [32]. This regional living community includes both rural regional living communities (rural communities) and urban regional living communities (urban communities). Urban communities are similar to what Tonnies calls "society", while rural communities correspond to "communities".

Compared with their urban counterparts, rural residents are more willing to help each other, even financially. They also tend to perceive a higher community cohesion and a larger supportive network size [33, 34]. On the other hand, urban residents generally are more likely to report foreseeable community threats such as public safety and environmental pollution than rural residents [35].

Additionally, China's urban–rural division system leads to differences in the management and quality of social services between urban and rural communities [36]. At the end of the 1950s, the ‘Regulations of the People's Republic of China on Household Registration’ were promulgated and implemented. It marked the emergence of a dual urban–rural division system in China, which remains influential to date [37]. According to the household registration system (*hukou*), every citizen is assigned to agricultural (rural) or non-agricultural (urban) groups based on the individual’s place of birth and parental *hukou* status [38, 39]. It is complicated to change *Hukou* status from rural to urban [39, 40]. As a determinant of privileges, hukou affects Chinese citizen’s socioeconomic well-being [40], resulting in disparities in economic development, access to medical services, education and job opportunities, retirement pensions, and mental health outcomes between urban and rural areas [38, 41–44]. For example, health care and medical insurance coverage are provided with higher quality in urban communities than in rural communities [45]. Most of the urban labor force participates in the Urban Employees Basic Medical Insurance (UEBMI). In contrast the rural labor force generally is covered in the New Rural Cooperative Medical System (NRCMS). The UEBMI is better off than NRCMS in terms of reimbursement thresholds and reimbursement ratios. In China, the overall level of medical benefits coverage ranked from high to low is the governmental employee healthcare system, the UEBMI, the Urban Residents Basic Medical Insurance (URBMI), the NRCMS [6].

Previous studies examined the urban–rural differences in community cohesion, supportive network size, foreseeable community threat, and medical insurance coverage among Chinese workers. However, they mainly used cross-sectional data. Additionally, the existing research failed to examine the long-term influence of community factors on depressive symptoms. Longitudinal studies are needed to shed light on the roles of different community factors within rural and urban contexts. Thus, this study fills the gap and identifies what community factors affect depressive symptoms in the labor force between urban and rural workers.

## Methods

### Study design

We used secondary data from the 2014 and 2016 China Labor-force Dynamics Survey (CLDS). The CLDS was a national representative longitudinal survey hosted by the Social Science Survey Center of Sun Yat-sen University, which cooperated with 28 universities in China to conduct the study and its follow-up survey in 29 provinces and cities, “excluding Hong Kong, Macao, Taiwan, Tibet, and Hainan” ([12] p. 1251). The CLDS used a multistage cluster, stratified, and PPS sampling method proportional to the size of the labor force.

First, 2,282 districts and counties from 29 provinces consistent with the sampling framework of the sixth census in 2010 were used as primary sampling units (PSU). Then, these districts were divided into six strata based upon geographic location (i.e., eastern, central and western regions) and population size (i.e., small and large). Next, in each stratum, all districts and counties were ranked according to the order of provinces, large cities, counties, small cities, and GDP levels. Then, systematic sampling approach was used to select PSU. In the selected PSU, streets and towns were ranked by their population size, they were then selected using systematic sampling as well. Selected streets and towns comprised the secondary sampling units (SSU). For each SSU selected, a household list was made from which 30 households were randomly as survey participants. Data was collected using the Computer Assisted Personal Interviewing (CAPI) as interviewers conducted face-to-face interviews.

Twenty-three thousand five hundred ninety-four individual labor questionnaires were collected in 2014, and 21,086 surveys were collected in 2016. This study included the answers from 9,140 participants (6,157 rural laborers and 2,983 urban laborers) who took part in the 2014 and 2016 CLDS.

### Measures

#### Dependent variable

The depressive symptoms were measured in the 2016 survey by using the Center for Epidemiological Studies Depression Scale (CES-D; [46]. It is a self-reported scale and consisted of 20 items measuring the frequency the participants experienced symptoms associated with depression over the past week. The scores on the scale ranged from 0 to 3 for each question: 0 = rarely or none of the time (less than one day), 1 = some or a little of the time (1–2 days), 2 = Occasionally or a moderate amount of the time (3–4 days), and 3 = Most or all of the time (5–7 days). Scores ranged from 0 to 60, with higher scores indicating more severe symptomatology. Cronbach's Alpha of the depressive symptoms factor was 0.945.

#### Independent variable–community factors

Community cohesion was measured by three questions: ‘How familiar are you with the neighborhood and other residents of this community (village)?’, ‘Do you trust the neighborhoods and other residents of this community (village)?’, and ‘Do you and your neighbors and other residents help each other?’. Each question is rated on a 5-point Likert response scale, ranging from 1 (very few) to 5 (very much). The total score ranged from 3 to 15, with a higher score indicating a higher level of community cohesion. The community cohesion’s Cronbach's Alpha was 0.799.

##### Community threat was measured via six questions on a four-point Likert scale

Questions asked the possibility of a community threat that would occur to participants in the next five years. They included "the likelihood of being unemployed, victimized by a criminal, attacked by a terrorist, affected by a contagious disease, subject to adulterated medications or food, and exposed to environmental pollution" ([6], p.320) . The total score ranged from 4 to 24 [6], with higher scores indicating a more foreseeable community threat. The Cronbach's Alpha of this scale was 0.831.

##### Community supportive network size

It was measured by four open-ended questions: among people that the participants feel close to in their local living community [12]: “1) how many they can ask for help; 2) how many they can share personal stories; 3) how many they can discuss important matters; 4) how many can lend them RMB 5000 (about $720) or above” ([12] p.1521). Then, none was coded as 1; 1–2 people was coded 2; 3–5 people was coded 3; 6–9 people was coded 4; and 10 and above people was coded as 5 [12]. The higher the cumulative scores of four questions indicated a larger community supportive network size. The Cronbach’s alpha of community supportive network size was 0.869.

##### Medical benefits coverage

As mentioned in the background, in China, the rank of medical benefits coverage is determined by the type of insurance. We used “1” to represent NRCMS, the lowest level of coverage and used “5” to represent government-supported medical, the highest level of coverage [6] . One score was added if any participant indicated having employee subsidized benefits or private insurance, and two scores were increased if they had both [6]. Therefore, medical coverage ranged from 1 to 7, with higher scores indicating higher levels of coverage.

### Control variables

Control variables included 2014 depressive symptoms and socio-demographic variables such as age and education. Different from the survey in 2016, the 2014 CLDS failed to use CES-D to evaluate depressive symptoms. We selected three questions from the 2014 depressive symptom measure as a baseline indicator of depressive symptoms. These three chosen questions asked participants during the past four weeks whether they “feel unhappy or upset”, “lose confidence in yourself”, and “feel unable to overcome the difficulties encountered”. The answers were collected by a 5-point Likert scale ranging from 1 (very few) to 5 (very much). Age was a continuous variable from 15 to 71. Educational level contained five categories: never attended school = 1; primary/private school = 2; middle school (junior high school, ordinary high school, vocational high school, technical school, technical secondary school) = 3; university (college, undergraduate) = 4; and master's degree and above = 5.

The participants were asked to rate their health condition on a 5-points Likert scale from 1 (very unhealthy) to 5 (very healthy). The self-rated class reflects the level of the participants’ status in politics, economics, education, etc. by asking the respondents to rate “their perceived social class on a Likert scale from 1 (lowest) to 10 (highest)” ([6], P. 321). Furthermore, a 5-point Likert scale, which was ranked from 1 (not satisfied at all) to 5 (very satisfied), was used to measure self-perceived job satisfaction [6].

### Analytical strategy

Descriptive analyses were conducted to summarize sample characteristics. Then, we used a t-test to ascertain the difference in community factors and depressive symptoms between urban and rural. We used two-tailed Pearson correlation analysis to examine the relationships between independent, control, and dependent variables. A hierarchical linear regression analysis was conducted to ascertain the influence of independent variables on the dependent variable. The equation below combined two levels of predictors of depressive symptoms.

$${y}_{i}={\beta }_{0}+ {\beta }_{1}{x}_{1i}^{^{\prime}}+{\beta }_{2}{x}_{2i}^{^{\prime}}+ {\varepsilon }_{i}$$

Y represents depressive symptoms and ${x}_{1}^{^{\prime}}$ represents a vector of individual-level control variables, including demographic factors, self-assessed health, self-assessed class, and self-perceived job satisfaction $.{x}_{2}^{^{\prime}}$ represents vectors of the second level community factors such as community supportive network size, foreseeable community threat, community cohesion, and medical benefits coverage. $\beta$ is a vector of coefficients associated with these observable characteristics that affect y. $\varepsilon$ refers to the idiosyncratic error. This model applied for both rural and urban workers in line with the research objectives.

The multicollinearity was ruled out through checking variance inflation factors (VIF) and tolerance scores. The results indicated that the tolerance  < 0.2 and VIF  > 5 of all variables. Thus, there was no concerns of multicollinearity. Additionally, the assumptions for the linear regression model were checked through the residual plots, and these assumptions for linear regression were met. We used the α level of  ≤ 0.05 as statistical significance in correlation analysis and regression analysis. All analyses were conducted using SPSS 24.0.

## Results

The overall characteristics of the participants and the t-test results are shown in Table 1. The T-test indicated that there were differences between urban and rural depressive symptoms and community factors. Rural labor reported more depressive symptoms than urban labor in 2016. The rural labor force had higher community cohesion compared to its urban counterpart. The results further revealed that education levels, self-rated health, self-rated class, and self-perceived job satisfaction were all significantly lower for the rural labor force. More so, the rural labor force had less foreseeable community threat, less medical insurance coverage, and smaller supportive network size compared to the urban labor force.

**Table 1:** Sample characteristics

	Rural workers(*N* = 6,157)	Urban workers(*N* = 2,983)	T test *p*-value
	M(SD)/ Percentage	M(SD)/ Percentage	
**Dependent variable**			
2016 Depressive symptoms	8.06(9.50)	6.40(8.32)	** < 0.001**
**Independent variable-Community Factors**			
Community cohesion	14.92(2.40)	13.03(2.64)	** < 0.001**
Foreseeable community threat	9.07(3.25)	10.44(3.65)	** < 0.001**
Supportive network size	2.78(1.12)	2.84(1.10)	**0.012**
Medical insurance coverage	1.19(0.69)	2.59(1.54)	** < 0.001**
**Control variable**			
Age	47.16(13.00)	42.98(12.66)	** < 0.001**
Education	2.47(0.99)	3.55(1.11)	** < 0.001**
no formal education	17.8%	4.1%	
elementary school	33.4%	12.5%	
middle school	35.3%	32.5%	
high school	11.0%	26.6%	
college and above	2.5%	24.4%	
Self-rated health	3.54(1.03)	3.78(0.90)	** < 0.001**
Self-rated class	4.42(1.66)	4.74(1.66)	** < 0.001**
Self-perceived job satisfaction	3.37(0.81)	3.43(0.78)	**0.002**
2014 Depressive symptoms	5.74(2.50)	5.88(2.42)	** < 0.001**

*Note*: Rural was coded as “1”; urban was coded as “2”

Table 2 shows correlation analysis between community factors and depressive symptoms of Chinese rural labor forces. Rural labor forces with more depressive symptoms in 2014, older, and less educated reported more depressive symptoms. Rural workers with poorer self-rated health, lower self-rated class, and lower self-perceived job satisfaction reported more depressive symptoms. At the community level, rural workers with more depressive symptoms also reported lower community cohesion, higher threat, less community support network, and lower medical insurance coverage.

**Table 2:** Correlation analysis of community factors and depressive symptoms of Chinese rural labor force

	2	3	4	5	6	7	8	9	10	2016 CES-D
1. 2014 depressive symptoms	-.022	-.100^b^	-.209^b^	-.173^b^	-.198^b^	-.157^b^	.098^b^	-.072^b^	-.062^b^	.234^b^
2. Age		-.405^b^	-.286^b^	-.049^b^	.058^b^	.146^b^	-.070^b^	-.021	-.054^b^	.095^b^
3. Education			.231^b^	.088^b^	.069^b^	-.041^b^	.034^b^	.121^b^	.221^a^	-.147^b^
4. Self-rated health				.244^b^	.105^b^	.118^b^	-.079^b^	.174^b^	.060^b^	-.268^b^
5. Self-rated class					.184^b^	.114^b^	-.126^b^	.154^b^	.064^b^	-.143^b^
6.Self-perceived job satisfaction						.104^b^	-.095^b^	.034^a^	.033^a^	-.071^b^
7. Community cohesion							-.175^b^	.330^b^	-.061^b^	-.053^b^
8.Community threat								-.090^b^	.012	.043^b^
9.Supportive network size									.034^b^	-.092^b^
10.Medical insurance coverage										-.074^b^

*Note*

^a^ Correlation is significant at the 0.05 level (2-tailed)

^b^ Correlation is significant at the 0.01 level (2-tailed)

Table 3 shows correlation analysis between community factors and depressive symptoms of Chinese urban labor forces. Table 3 shows that urban labor forces with more depressive symptoms in 2014 and less educated reported more depressive symptoms. Urban workers who had poorer self-rated health, lower self-rated class, and lower self-perceived job satisfaction reported more depressive symptoms. For community factors, urban workers with lower community cohesion, higher community threat, less community support network, and lower medical insurance coverage reported more depressive symptoms.

**Table 3:** Correlation analysis of community factors and depressive symptoms of Chinese urban labor force

	2	3	4	5	6	7	8	9	10	2016 CES-D
1. 2014 depressive symptoms	-.132^b^	.011	-.106^b^	-.095^b^	-.293^b^	-.143^b^	.199^b^	-.002	-.091^b^	.208^b^
2. Age		-.325^a^	-.263^b^	.023	.059^b^	.175^b^	-.133^b^	-.020	.113^b^	-.005
3. Education			.208^b^	.100^b^	.102^b^	-.160^b^	.064^b^	.173^b^	.382^b^	-.086^b^
4. Self-rated health				.261^b^	.115^b^	.082^b^	-.076^b^	.107^b^	-.038^a^	-.178^b^
5. Self-rated class					.181^b^	.150^b^	-.148^b^	.179^b^	.025	-.120^b^
6. Self-perceived job satisfaction						.115^b^	-.118^b^	.081^b^	.119^b^	-.119^b^
7. Community cohesion							-.140^b^	.180^b^	-.104^b^	-.063^b^
8.Community threat								.029	-.020	.068^b^
9.Supportive network size									.093^b^	-.094^b^
10. Medical insurance coverage										-.056^b^

*Note*

^a^ Correlation is significant at the 0.05 level (2-tailed)

^b^ Correlation is significant at the 0.01 level (2-tailed)

The results of the four regression models are shown in Tables 4 and 5. Table 4 shows regression analysis results of the influence of community factors on depressive symptoms of rural workers. Model 1 shows that depressive symptoms among the rural labor force in 2014 were significantly positively correlated with their depressive symptoms in 2016. It also shows that education level, self-rated class, and self-rated health predicted depressive symptoms two years later. Workers in rural areas who had less formal education, lower social class, and poorer self-rated health reported higher levels of depressive symptoms. Model 2 added the community factors. Lower medical insurance coverage predicted more depressive symptoms two years later, and the significance was approaching 0.05 level ( *p* = 0.056). Rural labor with lower medical insurance coverage reported more depressive symptoms.

**Table 4:** Regression analysis results of the influence of community factors on depressive symptoms of rural workers (*N* = 6,157)

	Mode1			Model 2		
	B	95%CI	*P*-value	B	95%CI	*P*-value
2014 Depressive symptoms	0.687	0.580, 0.793	**< 0.001**	0.683	0.576, 0.790	**< 0.001**
Age	0.010	-0.013, 0.034	0.384	0.012	-0.012, 0.036	0.335
Education	-0.761	-1.040, -0.482	**0.001**	-0.675	-0.964, -0.386	**< 0.001**
Self-rated health	-1.781	-2.048, -1.514	**< 0.001**	-1.746	-2.016, -1.475	**< 0.001**
Self-rated class	-0.291	-0.448, -0.134	**< 0.001**	-0.267	-0.426, -0.108	**0.001**
Self-perceived job satisfaction	0.010	-0.308, 0.327	0.952	0.009	-0.310, 0.327	0.958
Community cohesion				-0.010	-0.125, 0.105	0.867
Foreseeable community threat				0.001	-0.080, 0.081	0.986
Supportive network size				-0.195	-0.435, 0.045	0.112
Medical insurance coverage				-0.343	-0.695, 0.009	**0.056**
Adjusted R-square	0.111			0.111

**Table 5:** Regression analysis results of the influence of community factors on depressive symptoms of urban workers (*N* = 2,983)

	Mode3			Model 4		
	B	95%CI	*P*-value	B	95%CI	*P*-value
2014 Depressive symptoms	0.639	0.498, 0.779	** < 0.001**	0.639	0.496, 0.780	**< 0.001**
Age	-0.045	-0.076, -0.013	**0.006**	-0.045	-0.075, -0.010	**0.010**
Education	-0.514	-0.828, -0.201	**0.001**	-0.514	-0.781, -0.075	**0.018**
Self-rated health	-1.164	-1.546, -0.782	** < 0.001**	-1.164	-1.519, -0.746	**< 0.001**
Self-rated class	-0.223	-0.423, -0.023	**0.029**	-0.223	-0.370, 0.037	0.109
Self-perceived job satisfaction	-0.344	-0.776, 0.088	0.119	-0.344	-0.740, 0.127	0.165
Community cohesion				0.025	-0.105, 0.156	0.702
Foreseeable community threat				0.041	-0.050, 0.132	0.376
Supportive network size				-0.539	-0.842, -0.236	** < 0.001**
Medical insurance coverage				-0.017	-0.247, 0.213	0.882
Adjusted R-square	0.076			0.079		

Table 5 shows regression analysis results of the influence of control variables and independent variables on depressive symptoms of urban labor. Model 3 shows that for urban labor, the baseline depressive symptoms in 2014 were significantly positively correlated with depressive symptoms in 2016. Urban workers who were younger had lower education levels and had poorer self-rated health reported more depressive symptoms. Model 4 controls the variables of model 3. The change in adjusted R-square values from Model 3 to Model 4 was significant (*p* = 0.012). The results show that the community supportive network size significantly affected depressive symptoms of urban labor. Participants with smaller supportive network reported more depressive symptoms.

## Discussion

The study used longitudinal data to identify predictors for depressive symptoms among rural and urban workers respectively. Lower formal education, poorer self-rated health,, and lower self-rated class are socioeconomic and demographic factors predicting more depressive symptoms for both rural and urban workers. Differences were noted regarding the roles of studied community factors on laborers’ depressive symptoms. Depressive symptoms of rural workers were more affected by their medical insurance coverage, while depressive symptoms of urban labor were predicted by supportive network size. Specifically, rural laborers with lower medical insurance coverage predicted more depressive symptoms two years later Urban laborers with smaller community support sizes foresaw more depressive symptoms two years later.

Medical insurance coverage predicted rural workers’ depressive symptoms. The reason may be that the rural labor force is facing more pressure to pay for medical treatment [47]. For example, the medical insurance coverage and reimbursement scope in rural areas were significantly lower than those in urban China. Also, within rural population, there are also heterogeneities in terms of medical benefit coverage, which is closely tied to health and mental health care access. Lower medical insurance benefits suggested less security and more health risks, which can lead to poor mental health [48]. If the medical insurance coverage among the labor force improves, it can substantially improve their physical and mental health [49]. Still, the interpretation needs to be dealt with caution, given a borderline significance level identified.

The results also show that the supportive network size affects the depressive symptoms of the urban labor force. The possible reason is that the urban community environment is less emotional and trustworthy than the rural community, which makes supportive networks critically important [50, 51]. China has long been described as an “acquaintance society” [52], similar to the rural community concept of Tonnies, where interpersonal relationships are highly local and the mobility among community members is limited [53]. However, the relationship in urban community has become heterogeneous and complex due to social and economic structures associated with industrialization and modernization [53]. Urban workers may experience stress and pressure from these complicated relationships, which may trigger negative emotions and poor mental health when help is not available [30, 54]. Thus, the available supportive network is a critical factor affecting urban workers’ depressive symptoms. Thus, empathy and support from informal networks can help urban laborers effectively manage their negative emotions [55]. This finding largely corresponds to the Tonnies’ theory of differences in the rural and urban communities.

The influence of foreseeable community threat and community cohesion on laborers' depressive symptoms was not significant in either rural or urban settings. Previous research using cross-sectional data found that community cohesion could alleviate depressive symptoms [56, 57]. However, our study, which used the longitudinal data, indicated that community cohesion could not predict depressive symptoms. This may be because individuals have adapted to the community environment as they reside longer in their community [23]. Therefore, community cohesion and community threat at baseline fail to predict individual emotions over time. This finding also shows the need to examine the relationships among community cohesion, community threat and people's emotion wellbeing longitudinally. Several limitations inherent to this study need to be noted. First, the number of rural worker respondents is more than their counterparts. This largely reflected a social fact of the population distribution in China: the number of households in the rural community is more than in urban communities. However, the unbalanced sample would benefit from adjustment using weighting methods if weighting procedures had been adopted in the sampling stages. Future research needs to balance the number of urban and rural labor forces for comparison. Second, the time period covered by the study and, above all, the fact that they are "pre-COVID" data. Therefore, the clinical implications of this study after the COVID-19 need further discussion. Lastly, the 2014 CLDS failed to use the CES-D to evaluate depressive symptoms. As mentioned in the method part, we selected three questions that best represent depressive symptoms from the 2014 CLDS as a baseline control variable to solve this problem.

### Implications for policy and practice

The results further indicate that the rural labor force has more depressive symptoms than their urban counterparts. The roles of community factors on the depressive symptoms manifested differently among the urban and rural labor force. The results provide empirical data to understand the various factors affecting the depressive symptoms of China's urban and rural labor force. Also, they present policy suggestions for China's urban and rural governance and community construction. In the development of rural communities, it is crucial to reduce the gap between urban and rural medical insurance coverage. The covered benefits of NRCMS remained lower than the three-tier healthcare coverage (public healthcare, UEBMI, & URBMI) available in urban areas  [58]. The NRCMS “needs to be improved in many aspects, such as hospital and clinic accessibility, covered diseases, drug benefits, copayments and deductibles” ([6], pp. 323). Additionally, to further reduce the rural–urban disparities in depressive symptoms, the government should also “aim to eradicate the structural segmentation rooted in the current household registration system that resulted in the fragmentation of the medical insurance system” ([6], pp. 323).

Furthermore, urban community governance may consider organizing inclusive community activities and broadening the supportive network size of the urban labor force. Thus, urban laborers may build a supportive network that can seek help, share their minds, and relieve stress. Ultimately, the supportive system may mitigate the depressive symptoms of the urban labor force due to work pressure. The “quasi-acquaintance community” model tested in Shenzhen, a well-developed city next to Hong Kong in southern China, has positively increased community support among urban residents [59]. Other cities may learn from the “Shenzhen model” and design solutions based on their cities' historical, social, and economic factors.

## Conclusions

This study affirmed that rural laborers had higher depressive symptoms than their urban counterparts and suggested different predictors for depressive symptoms between urban and rural workforce. Medical benefits coverage predicts depressive symptoms of rural workforces, while community supportive network size affects depressive symptoms of urban workforces. The different predictors for urban and rural workforces call for strategically designed policy and practice to be in place to address depressive symptoms in the workforce living in urban and rural Chinese communities.

## Data Availability

The datasets used and/or analysed during the current study are available from the Social Science Research Center of Sun-Yatsen University on reasonable request.

## Abbreviations

CES-D: Center for Epidemiological Studies-Depression
CLDS: China Labor-force Dynamics Survey
CAPI: Computer Assisted Personal Interviewing
NRCMS: New Rural Cooperative Medical System
PSU: Primary Sampling Units
SSU: Secondary Sampling Units
